# Highly Sensitive Temperature Sensing in Biological Region with Ratiometric Fluorescent Response

**DOI:** 10.3390/molecules30051121

**Published:** 2025-02-28

**Authors:** Yan Li, Han Yu, Hongjuan Li, Shiguo Sun, Ruijin Yu, Yongqian Xu

**Affiliations:** College of Chemistry & Pharmacy, Northwest A&F University, Yangling 712100, China; q1270572829@163.com (Y.L.); yher.x@nwsuaf.edu.cn (H.Y.); hongjuanli@nwafu.edu.cn (H.L.); sunsg@nwsuaf.edu.cn (S.S.)

**Keywords:** ratiometric fluorescence, temperature sensors, Poly(2-oxazoline)

## Abstract

Poly(2-oxazoline) (POx), a typical thermoresponsive polymer with good biocompatibility, was conjugated with environment-sensitive tetraphenylenethene (TPE) and hydroxyphenylbenzoxazole (HBO) to achieve unique thermometer readings. Through phase transition induced by temperature, the thermometers can measure temperature in biologic range with ratiometric fluorescence response, ultrahigh sensitivity and good reversibility. Moreover, the thermometer can be used to measure the change in temperature with large fluorescence difference in living cells.

## 1. Introduction

Temperature is a fundamental physical parameter in biology and medical science, closely linked to a wide range of biological activities, such as intracellular chemical reactions and biological processes including enzymatic reactions, gene expression, cell division, and molecular interactions [1]. Research has shown that abnormal temperature variations are often associated with a range of pathological changes, with inflammation and tumorigenesis being characterized by elevated temperature [2]. Detecting intracellular temperature can enhance the understanding of cell biology and provide valuable insights for disease diagnosis and therapy. Thus, it is essential and significant to develop techniques and methods capable of accurately detecting temperature within biological systems [3].

Although the traditional thermocouple method can rapidly and accurately measure the intracellular temperature, it is limited by cell damage and low spatial resolution [4,5,6]. Alternatively, fluorescence thermometers with fluorescence microscopy capability, simple operation, high sensitivity, spatiotemporal resolution, noninvasiveness, as well as potential applications in living cells have been developed [7,8,9].

Considering the practical application in complex biological environments, an ideal thermometer should exhibit the following features. First, the fluorescence intensity change in response to temperature variations should be sufficiently large to ensure high sensitivity, enabling precise measurement of small temperature changes. Second, the thermometer should have high stability against photoexcitation and cellular materials, as well as excellent temperature-responsive reversibility. Third, it should be biocompatible and nontoxic, critical features for long-term studies in biologic systems. Last but not least, it is preferable that the fluorescence reading from thermometer be ratiometric [10,11,12,13]. Up to now, researchers have developed various types of ratiometric fluorescent thermometers based on different materials: (i) Organic fluorophores, such as Rhodamine B (RhB) and triarylamine derivatives, which achieve ratiometric responses through intramolecular charge transfer (ICT) mechanisms [14,15]. (ii) d-Block metal complexes, exemplified by Eu(II) coordination complexes, which exploit ligand field-sensitive variations in luminescence lifetimes for temperature sensing [16,17]. (iii) f-Element nanomaterials, including Er^3^⁺/Yb^3^⁺ co-doped systems [18], which leverage thermally coupled energy levels. At varying temperatures, thermal excitation of the energy levels induces shifts in fluorescence emission intensities, enabling self-referenced temperature measurement. (iv) Quantum dots (QDs): Within specific temperature ranges, the bandgap structure of QDs undergoes temperature-dependent alterations, modulating their fluorescence properties. This size-dependent fluorescence signature facilitates temperature quantification [19,20]. (v) Metal–organic frameworks (MOFs): Owing to their high specific surface area and tunable pore architectures, MOFs allow efficient encapsulation of fluorescent probes, thereby enabling ratiometric temperature detection [21,22]. Although these systems each have their own unique features, they often require the cooperation of multiple components or complex wavelength modulation to achieve ratiometric detection. Moreover, the potential risk of cytotoxicity limits their practical application in living cells. To date, most thermometers rely on monitoring fluorescent intensity change at a single wavelength, which poses problems and limitations for accurate measurement due to influences from thermometer concentration, autofluorescence and instrumental fluctuation. Ratiometric thermometers, which measure the ratio of fluorescence intensities at two different wavelengths can effectively eliminate these effects, thereby meeting the requirement for precise detection [23,24].

Organic dye molecules, such as Rhodamine B (RhB) and pyrene derivatives, revealing temperature-responsive characteristics, have been used for temperature sensing of biological samples in fluids; however, they suffer from lower sensitivity. Combining them with temperature-sensitive polymer can solve this issue [25,26]. The temperature-sensitive polymer transforms the temperature change into alterations in the external environment (such as polarity) of the fluorescent dyes, while many fluorescent dyes are sensitive to the surrounding environment [27]. By employing this strategy, the fluorescence alteration induced by temperature is effectively amplified, resulting in the achievement of high sensitivity [28]. The thermometers with high degree of fluorescence change and good water solubility significantly improve the wide application in microenvironmens, such as intracellular measurement of temperature [29].

Poly(2-oxazoline) (POx), a typical thermoresponsive polymer exhibiting the Lower Critical Solution Temperature (LCST) around body temperature, is biocompatible, biodegradable, and possesses stealth characteristics both in vitro and in vivo [30,31,32]. POx has been investigated as an alternative to poly(ethylene glycol) (PEG). The biocompatible characteristics of POx provide promising applications in biological systems. In addition, POx offers several additional advantages such as higher stability, lower viscosity, and facial synthesis. Jang et al. incorporated pyrene, BODIPY and porphyrin fluorophores into Pox [33]. Through their combination, temperature responsive ratiometric and even white emission were achieved [34].A ratiometric fluorescence response from this combination of multiple dyes can only be obtained after strict and complicated tuning of dye concentration ratio [35], which limits the practical applications in intracellular measurement. Therefore, it is necessary to develop a new single fluorophore to hybrid with POx to realize temperature sensing with ratiometric response. TPE is an environment-sensitive fluorophore. In its solution state, the fluorescence intensity of uncontrolled TPE is very weak at 380 nm due to the rapid relaxation by the motion of phenyl rings. However, in its aggregates, the motion of phenyl rings is restricted, producing strong aggregation induced emission (AIE) at 470 nm [36]. HBO possesses excited-state intramolecular proton transfer (ESIPT) properties, rendering dual emissions from its excited *enol* and *keto* tautomers [37,38]. The ratio of dual emissions intensities depends on ESIPT process which is highly susceptible to external environment (such as intermolecular hydrogen bonding).

**Scheme 1:**
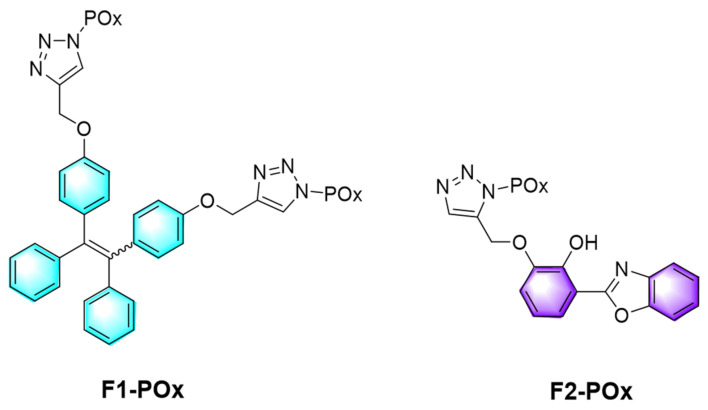
The structures of F1-POx and F2-POx.

In this work, we designed two kinds of POx-based hybrids F1-POx and F2-POx, where tetraphenylenethene (TPE) and hydroxyphenylbenzoxazole (HBO) were introduced into POx through a copper-catalyzed azide-alkynyl cycloaddition (CuAAC) coupling reaction, respectively (Scheme 1). It is envisioned that F1-POx and F2-POx will produce ratiometric fluorescence signal upon temperature change because the thermoresponsive POx segment of F1-POx and F2-POx undergo phase transition process as temperature varies. To the best of our knowledge, this is the first study to report POx-based thermometers with appended a single fluorophore that can produce ratiometric fluorescence change for temperature sensing. Compared with the previously reported ratiometric temperature probes, F1-POx and F2-POx offer several additional advantages, such as excellent biocompatibility, good photostability, supra-high sensitivity and favorable reversibility.

## 2. Results and Discussion

The synthesis of F1-POx and F2-POx is shown in Supporting Information. Briefly, azide-bearing POx were prepared through living cationic polymerization of 2-isopropyl-2-oxazoline monomer initiated by methyl *p*-tosylate as the initiator and terminated by sodium azide. Then, functional POx further reacts with propargyl-conjugated fluorophores through CuAAC coupling reaction to offer corresponding compounds.

**Figure 1:**
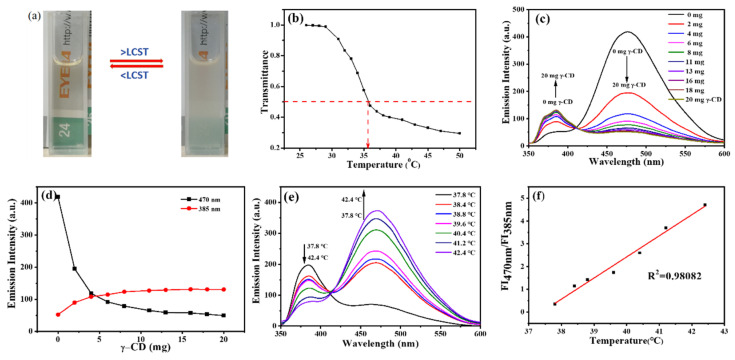
(**a**) Photographs of F1-POx solutions in water at 25 °C (left) and 50 °C (right). (**b**) The spectra of transmittance with increasing temperature for F1-POx in water (10 μM). (**c**) Fluorescence spectra of F1-POx in PBS buffer (10 mM, pH 7.4) upon addition of γ-CD (0–20 mg), λ_ex_ = 305 nm. (**d**) The fluorescence intensity changes at 470 nm (black) and 385 nm (red) of F1-POx in PBS buffer (10 mM, pH 7.4) upon addition of γ-CD (0–20 mg). (**e**) Fluorescence spectra of F1-POx with 10 equivalent of γ-CD in PBS buffer (10 mM, pH 7.4) at various temperatures, λ_ex_ = 305 nm. (**f**) The plot of fluorescence intensity (at 470 nm and 385 nm) ratio changes in F1-POx to temperature changing from 33 to 41 °C.

First, we studied the fluorescence spectra of the sensor F1-POx in aqueous solution. As shown in Figure S5, F1-POx exhibits **a** distinct emission peak at 470 nm, which is an AIE characteristic peak of TPE units. The fluorescence intensity of this peak obviously decreased with increasing of temperature (Figure S5a). There is a remarkable response within the temperature range of 27–48 °C. We further measured the fluorescence spectra change in F1-POx at interval of 1 °C from 31.8 to 39.6 °C (Figure S5c). Unfortunately, there is no obvious linear relationship between the fluorescence intensity at 470 nm and the temperature in this range (Figure S5d). The LCST value of the F1-POx sensor was determined to be approximately 36 °C based on the transmittance change curve of the solution as temperature varied (Figure 1a,b). Typically, the aqueous solution undergoes a characteristic phase transformation near the LCST point, resulting in a large deviation, which is likely due to the nonlinear fluorescence response. To optimize the performance of F1-POx for temperature sensing, a host–guest interaction was introduced into this system to reduce or avoid this large deviation in which temperature is close to LCST. TPE units in F1-POx are hydrophobic, which could be accommodated in the cavity of γ-cyclodextrin (γ-CD) [39]. The fluorescence intensity of F1-POx upon the addition of γ-CD was monitored. As shown in Figure 1c, the fluorescence intensity at 470 nm gradually decreased, and the fluorescence intensity at 380 nm assigned to TPE monomer increased accompanied with an isosemission point at 411 nm, suggesting that TPE units in F1-POx formed a host–guest complex with γ-CD. In addition, cyclodextrin inhibited the aggregation of F1-POx near the LCST (Figure S6). The motion of TPE units in γ-CD becomes free, resulting in the dissociation of aggregated state of the TPE units. The remarkable ratiometric fluorescence change triggered by the host–guest interaction improves the performance of F1-POx for temperature sensing.

**Figure 2:**
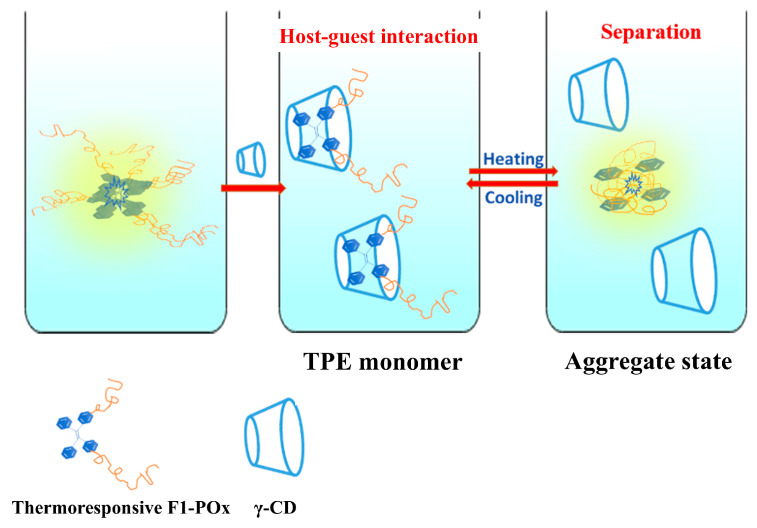
Schematic diagram of sensor F1-POx after heating and cooling.

The variation in fluorescence spectra of F1-POx in the presence of γ-CD as a function of increasing temperature was systematically investigated. The fluorescence intensity at 470 nm gradually increased, accompanied with a decrease at 385 nm, and an obvious ratiometric fluorescence response was observed (Figure 1e). There is a good linear relationship between the ratiometric fluorescence (*F*_470_/*F*_385_) and the temperature in range of 37.8–42.4 °C (Figure 1f). According to the linear relationship, the sensitivity for temperature was calculated as high as 274%/°C. To our best knowledge, the highest sensitivity of all reported thermometers is about 38%/°C [40]. The sensitivity of F1-POx with γ-CD is nearly 7-fold higher than the data. In addition, the solution showed fully reversible ratiometric fluorescence change without any attenuation after four sets of temperature change (Figure S7). The above results may be due to the hydrophilic POx chains becoming hydrophobic with increasing temperature. Correspondingly, the hydrophobicity of whole molecule of F1-POx would increase, resulting in the discharge of γ-CD from the host–guest complex (Figure 2).

**Figure 3:**
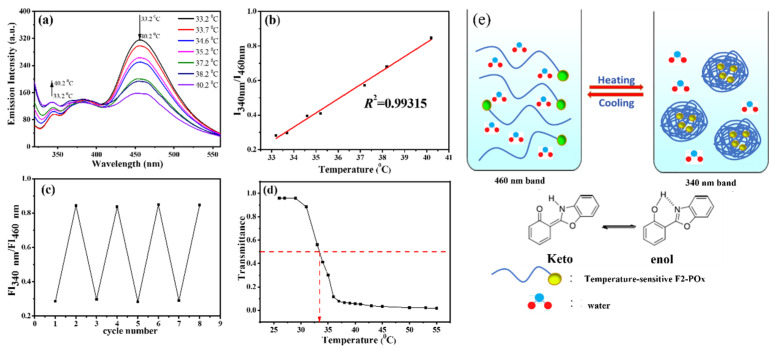
(**a**) Fluorescence spectra change in F2-POx in PBS buffer (10 mM, pH 7.4) with different temperatures, λ_ex_ = 305 nm. (**b**) The plot of fluorescence intensity (at 470 nm and 385 nm) ratio changes of F2-POx to temperature changing from 33 to 41 °C (**c**) The cycle number evaluation of fluorescence intensity (at 470 nm and 385 nm) ratio of F2-POx vs. the repeated heating and cooling cycles at 33 °C and 40 °C. (**d**) The spectra of transmittance with increasing temperature for F2-POx in water (10 μM). (**e**) Schematic diagram of sensor F2-POx after heating and cooling, and the structures of enol-keto tautomers of HBO.

F2-POx showed two emission peaks at 340 and 460 nm, which are attributed to the normal isomer (N* enol emission) and tautomer (T* keto emission) of HBO units, respectively [41]. With increasing temperature, the intensity of the enol emission gradually increased, while the keto emission intensity at 460 nm decreased (Figure 3a). A good linear relationship between the ratiometric fluorescence (F340/F460) and temperature was observed in the range of 33.2–40.2 °C (Figure 3b), demonstrating the potential applicability in biological systems close to 37 °C. The sensitivity was calculated to be approximately 28.6%/°C. The ratiometric fluorescence could be fully recovered without any change after four cycles of heating and cooling, indicating excellent reversibility for temperature sensing (Figure 3c). ESIPT process of HBO moiety producing keto emission is sensitive to environment changes. The LCST value of F2-POx is 34 °C (Figure 3d). As the temperature goes up, F2-POx would undergo phase transition, and the weak hydrogen bonding interactions between HBO units and water would switch to the relatively strong intra- and/or intermolecular ones between HBO units and POx chains, resulting in the ratiometric fluorescence response (Figure 3e).

**Figure 4:**
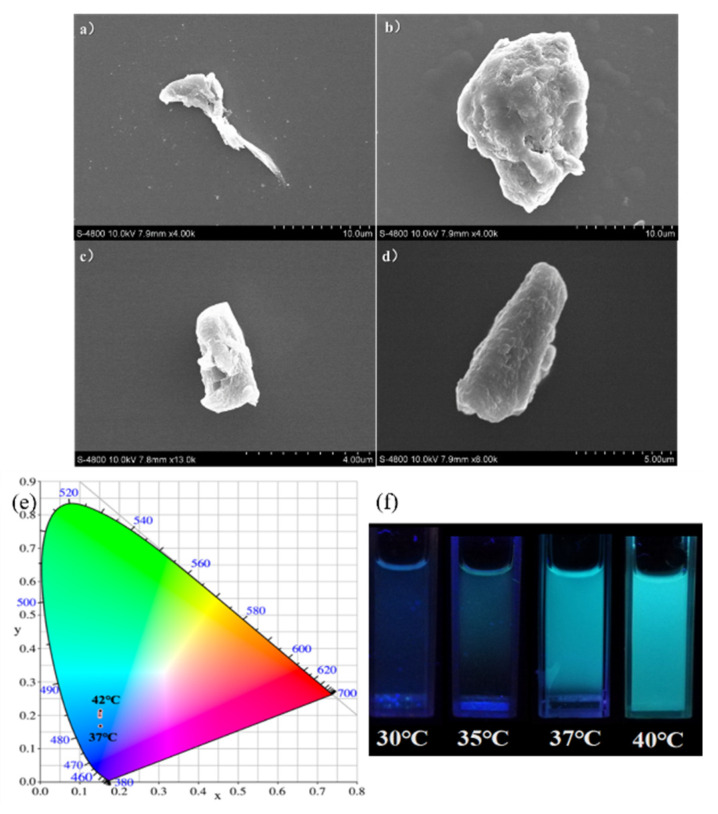
SEM image of sensor F1-POx in aqueous solution at (**a**) room temperature, (**b**) 40 °C, (**c**) room temperature with excess γ-CD, (**d**) 40 °C with excess γ-CD. (**e**) CIE chromaticity diagram showing the (x, y) color coordinates of F1-POx in the presence of γ-CD (20mg) as a function of temperature. (**f**) Photographs of aqueous solutions of F1-POx in the presence of γ-CD (20mg) at different temperatures, excited using a 365 nm UV handheld lamp.

To gain insight into the phase transition process, scanning electron microscope (SEM) images of F1-POx in the absence and presence of γ-CD before and after heating were investigated. As the temperature increases, the hydrophilic POx chains become hydrophobic, causing the size of the thermometer to increase, which demonstrates aggregation occurs due to the phase transition process. (Figure 4a–d). The color change in F1-POx solution with increasing temperature can be observed through CIE chromaticity diagram which is independent of experimental instrument and describes the colors by mathematical calculation. As shown in Figure 4e,f, the fluorescence color changes from blue-purple to blue-indigo with the increase in temperature. This color change in the solution can also be seen by naked eyes.

**Figure 5:**
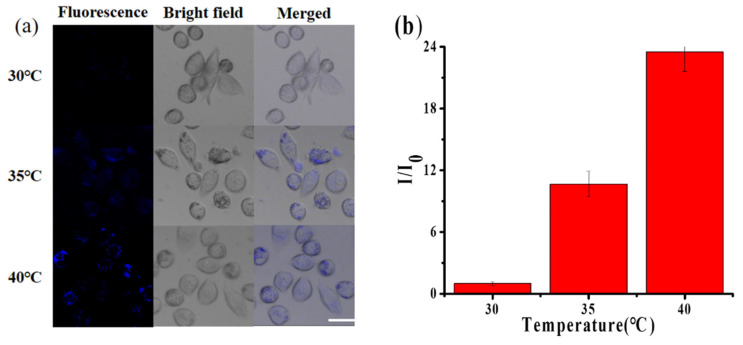
(**a**) Confocal fluorescence images of liver cancer cells (HepG2 cells). Images were acquired by using excitation wavelength of λ_ex_ = 405 nm. Scare bar: 20 µm. (**b**) Fluorescence intensity histogram in cells, error bars standard for the mean value of three experiments.

The F1-POx demonstrates sensitive temperature response performance with potential for temperature sensing. Given the good biocompatibility and no toxicity of POx, F1-POx was further applied for temperature sensing in living cells. HepG2 cells were incubated with 10 μM F1-POx. After incubation at 37 °C for 1 h, the images were observed by laser confocal microscopy. As shown in Figure 5, the fluorescence intensity of cells increases obviously with the increase in temperature. As temperature increases from 30 to 40 °C, a ten-fold enhancement of fluorescence was found, suggesting the potential practical application of temperature sensing in living cells.

## 3. Materials and Methods

### 3.1. General Procedure for Analysis

The sensor F1-POx and F2-POx were dissolved in acetonitrile, and the concentrations were fixed to 0.01 mol/L. During the test, the solutions were diluted with phosphate buffer (PBS, pH = 7.4, 10 mM) to required concentration. Temperature control and measurement were as follows: the tested solutions were put in water bath with fixed temperature, stirred for 10 min to ensure the temperature of solutions is constant, and the temperature was accurately measured with an infrared thermometer. In order to reduce temperature change during the process of experiments, the measurement is rapid and the temperature value was rechecked again after measurement.

### 3.2. Evaluation of Performance of Temperature Sensitive Sensors

For fluorescent temperature sensors, the temperature sensitive performance is evaluated by the corresponding relationship between the fluorescence intensity and the temperature. Usually, three parameters including measurement range, sensitivity and repeatability are used to evaluate the temperature sensitive performance of the sensor.

The measurement range indicates the temperature range corresponding to the obvious response of the fluorescence spectra of the sensor. In an ideal scenario, a larger temperature range would indicate better performance. But in practice application, the temperature range that can meet the measurement conditions is most important. Sensitivity is a crucial metric for characterizing a sensor’s ability to detect temperature changes. The sensitivity of the fluorescence temperature sensor can be expressed by enhancement of the fluorescence intensity when the temperature changes to a certain extent. The sensitivity is divided into absolute sensitivity and relative sensitivity. The absolute sensitivity is defined as the change in fluorescence intensity when the temperature changes up to 1 °C. The relative sensitivity is defined as the percentage of the change in fluorescence intensity relative to that of itself when the temperature changes up to 1 °C. Due to the influence of experimental conditions, absolute sensitivity is not always suitable for characterizing the temperature sensitivity of fluorescence temperature sensors. Therefore, relative sensitivity is chosen to evaluate the sensitivity of the sensor. For the system with linear relationship between fluorescence intensity and temperature, the relative sensitivity can be carried out by the following formula [40]:

$$S_{re}=\left|\frac{I-I_{0}}{I_{0}\Delta T}\right|$$

where I is the fluorescence intensity of thermometer at certain temperature T, I_0_ is the fluorescence intensity of thermometer at certain temperature T_0_, ΔT is T − T_0_.

In order to be applied for practical application, besides excellent temperature responsiveness and sensitivity, the sensor should have good reversibility (i.e., the recoverability and reproducibility of fluorescence after heating and cooling). The reversibility of the sensor system is evaluated by measuring the fluorescence spectra of the sensor system at two temperatures for many times. If the reversibility is good, the fluorescence intensity of the sensor should not change or change very little after repeated measurement.

### 3.3. Measurement of LCST Value of Solution with Sensors

Phase change polymers with LCST are among the most widely studied thermal responsive polymer materials. Polymer POx is a typical LCST typed polymer. LCST is defined as the lowest critical solution temperature. When the aqueous solution temperature is below the LCST, the polymer chain is completely dissolved by hydrogen bonding, and the system is usually clear and transparent. When the temperature exceeds the LCST, the hydrogen bonds are destroyed, causing the polymer chains to dehydrate and form stable micelles, making the solution turbid with decreased transmittance. However, the system can return to the original state after cooling. LCST value is one of the important and basic parameters of this kind of polymer [42]. Firstly, the phase transition phenomena of aqueous solution containing sensor with temperature change are observed. Then, the LCST value can be measured by the ultraviolet absorption spectra of the aqueous solution with polymer. The LCST value can then be determined by analyzing the ultraviolet absorption spectra of the aqueous solution. A transmittance change curve is obtained as the temperature is varied, and the corresponding temperature at which transmittance reaches 50% is defined as the LCST value. The LCST value for the sensor F1-POx is found to be approximately 36 °C, while the LCST for the sensor F2-POx is around 34 °C (the intersection point of the dotted line and abscissa in the diagram is LCST value). This is slightly different from the LCST value of the single POx molecule, which is around 37 °C. The difference is presumed to be caused by the different fluorescent molecules.

Other relevant experimental conditions are listed in the Supplementary Materials.

## 4. Conclusions

In conclusion, two types of thermometers were synthesized, incorporating single and environmental fluorophores with thermoresponsive, biocompatible, and non-toxic POx. These thermometers exhibit a phase transition induced by the switch from hydrophilicity to hydrophobicity, enabling them to produce ratiometric fluorescence responses to temperature changes. They can measure temperatures within the biological range with exceptional sensitivity and good reversibility. The temperature-dependent fluorescence change can be detected through naked eyes. Furthermore, the thermometer can be used to measure the change in temperature in living cells. The unique polymer-based thermometers hold great potential for studying and diagnosing temperature-related biologic processes and diseases.

## Data Availability

Data are contained within the article.

## Supplementary Materials

The following supporting information can be downloaded at: https://www.mdpi.com/article/10.3390/molecules30051121/s1. Figure S1. The synthetic routine for POx. Figure S2. The synthetic routine for F1-POx. Figure S3. The synthetic routine for F2-POx. Figure S4. Analysis of the GPC chromatogram for F1-Pox. Figure S5. (a,c) Fluorescence spectra of F1-POx in PBS buffer (10 mM, pH 7.4) at various temperatures, λex = 305 nm, the temperature increases at 2–3 °C (a) and 0.6–1.4 °C (b) intervals; (b,d) Fluorescence intensity of F1-POx at 470 nm vs temperature in PBS buffer (10 mM, pH 7.4), here (b) and (d) are corresponding to (a) and (c), respectively. Figure S6. Photos of F1-POx in aqueous solution in the presence of cyclodextrin at 25 °C (left) and 50 °C (right). Figure S7. Fluorescence intensity ratio changes of F1-POx and excess γ-CD upon repeated heating and cooling cycles at 36 °C and 41 °C. Figure S8. 1H NMR spectrum of 1 in DMSO-d6. Figure S9. 1H NMR spectrum of F1 in DMSO-d6. Figure S10. 13C NMR spectrum of F1 in DMSO-d6. Figure S11. 1H NMR spectrum of Ox in CDCl_3_. Figure S12. 1H NMR spectrum of POx in CDCl_3_. Figure S13. 1H NMR spectrum of 3 in DMSO-d6. Figure S14 1H NMR spectrum of F2 in CDCl_3_. Figure S15. 13C NMR spectrum of F2 in DMSO-d6. Refs. [43,44,45,46,47,48] are cited in the Supplementary Materials.
