# Supplementary material for: Highly Sensitive Temperature Sensing in Biological Region with Ratiometric Fluorescent Response
Source: Molecules. 2025 Feb 28;30(5):1121. doi: 10.3390/molecules30051121 (PMC11902262; doi:10.3390/molecules30051121)
Supplement: Supplementary file 1 [file molecules-30-01121-s001.zip › molecules-3443778-supplementary-final version.pdf]

Supplementary Materials

Highly sensitive temperature sensing in biological region with ratiometric fluorescent response

Yan Li, Han Yu, Hongjuan Li, Shiguo Sun, Ruijin Yu* and Yongqian Xu*

Shaanxi Key Laboratory of Natural Products & Chemical Biology, College of Chemistry & Pharmacy, Northwest A&F University, Yangling, Shaanxi, 712100, P.R. China.

1. Experimental section

1.1 Materials and equipment

All chemicals were purchased from Aladdin and Mayer Chem. Reagents Co. (Shanghai, China) and Wolsen Bio. Reagents Co. (Xiaan, China). All chemicals and reagents were used directly as obtained commercially unless otherwise noted. ^{1}H spectra were recorded on a Bruker AVANCE III 500 or 400 spectrometer (Germany) with the use of TMS as internal standard. Absorption and emission spectra were collected by using a Shimadzu 1750 UV-Visible spectrometer and a RF-5301 fluorescence spectrometer (Japan), respectively. Cell images were acquired by utilizing Olympus fluorescence microscopy and FX Pro (Leica TCS SP8, Germany).

1.2 Experiments for cell imaging

Human hepatoma cells (HepG2) were cultured with 1640 (10% fetal bovine serum, 50 U mL^{-1} penicillin and 50 U mL^{-1} streptomycin), DMEM (Dulbecco's modified Eagle's medium), 10% fetal bovine serum, 50 U mL^{-1} penicillin and 50 U mL^{-1} streptomycin) in a 37 ^{0}C incubator containing 5% CO_2.

When the cells reached the stage of logarithmic growth, the HepG2 cells were washed with PBS buffer three times, and then digested with 0.25% (v/v) trypsin at 37

* E-mail addresses: yuruijin@nwsuaf.edu.cn (Ruijin Yu); xuyq@nwsuaf.edu.cn (Yongqian Xu)

^{0}C for 1 min. The upper trypsin was carefully sucked out. The solution of cell suspension was prepared by blowing and mixing the solution. The solution was centrifuged at 1000 rpm for 5 min, washed twice with PBS buffer solution, and incubated into a culture dish for imaging experiments.

The probe **F1-POx** was incubated with HepG2 cells for 1 h, and then the cells were washed with PBS buffer three times to remove the free dyes that did not enter the cells. Laser confocal microscopy was used for the imaging experiments. The excitation source was set up at 405 nm and all pictures were taken at the same parameters to minimize possible errors.

2. Synthesis of compound

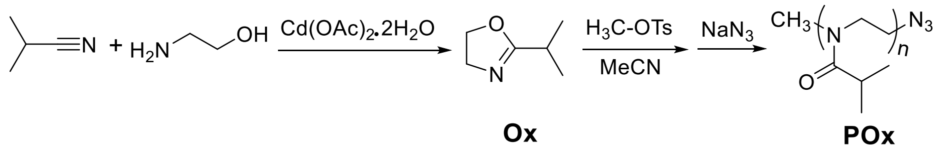

Fig. S1 The synthetic routine for **POx**.

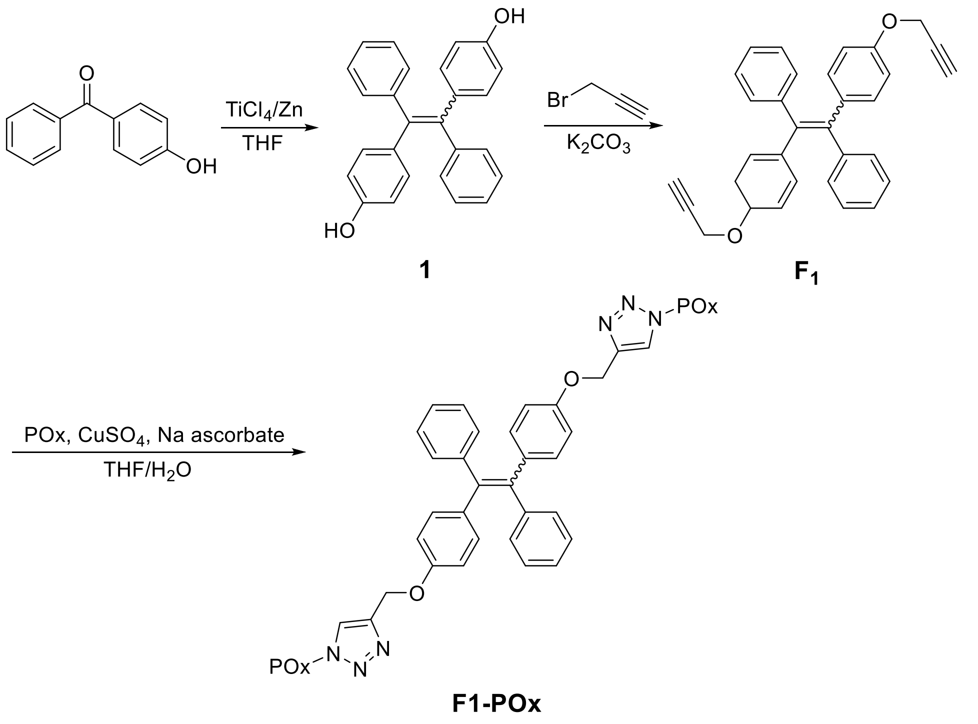

Fig. S2. The synthetic routine for **F1-POx**.

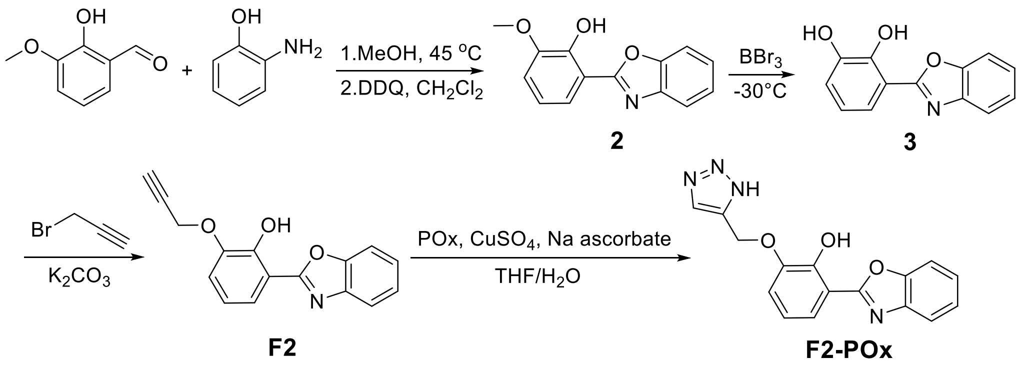

Fig. S3. The synthetic routine for **F2-POx**.

2.1 Synthesis of compound 1

The procedure of synthesis was conducted as previously reported.[S3] Zinc powder (2.9 g, 44 mmol) and 4-hydroxybenzophenone (2.0 g, 10 mmol) were mixed into a 250 ml round-bottom flask with a reflux condenser. After adding 100 mL of anhydrous THF, the air in the flask was replaced with nitrogen. The mixture was cooled in an ice-salt bath to -10 ^{0}C, and $TiCl_4$ (2.5 mL, 22 mmol) was slowly injected into the reaction solution using a syringe. The mixture was slowly heated to room temperature, stirring for 0.5 h and then heated up overnight. After the reaction was completed, the reaction solution was quenched by 10% K_2CO_3 aqueous solution (slowly dropping until bubbles were no longer produced). The mixture was extracted with ether three times, washed with saturated NaCl aqueous solution two times, and dried with anhydrous sodium sulfate. After the solvent was dried, the residue was purified by silica gel column with petroleum ether/ethyl acetate (v/v 10:3) as an eluent. The 1.1 g white solid was obtained and the yield was 60.4%. The following were obtained: ^{1}H NMR (500 MHz, DMSO-d_6) δ ppm, 9.28 (d, J = 12.0 Hz, 2H), 6.99 (ddd, J = 8.8, 9.3, 6.8 Hz, 10H), 6.69 (dd, J = 8.2, 7.9 Hz, 4H), and 6.47 (dd, J = 11.4, 7.8 Hz, 4H).

2.2 Synthesis of compound F1

The synthetic procedure was followed as reported previously in the literature.[S4] TPE-OH (0.3 g, 0.82 mmol) and anhydrous potassium carbonate (0.87 g, 6.3 mmol) were placed in a 100 mL single-neck round-bottom flask, and into which 30 mL of anhydrous acetone was added, stirred at room temperature for 1 h, dripped with 3-bromopropyne (220 mL, 2 mmol), and then heated up to reflux overnight. The residue was purified by silica gel column chromatography (petroleum ether: ethyl acetate = 20:1). A total amount of 0.16 g yellow slurry product was achieved and the yield was 44.3%. The following were obtained: ^{1}H NMR (500 MHz, $CDCl_3$): δ ppm, 7.12-7.07 (m, 10H), 6.99 (m, 4H), 6.74 (m, 4H), 4.60 (m, 4H), and 2.51 (m, 2H). ^{13}C NMR (126 MHz, DMSO-d_6) δ (ppm): 156.21, 144.11, 139.89, 136.89, 126.80, 139.80, 132.34, 131.21, 131.19,

126.35, 126.89, 114.62, 114.52, 79.70, 78.65, and 55.80.

2.3 Synthesis of compound Ox

The synthetic procedure was followed as reported previously in the literature.[S5] First, 2-aminoethanol (20 mL, 0.334 mol) was added dropwise to isobutyronitrile (28.3 mL, 0.316 mol) solution containing cadmium acetate dihydrate (4.26 g, 0.016 mol) and stirred for 24 h. After reaction, the product was purified by distillation, and the fraction near 120 ^{0}C was collected. The colorless liquid product was obtained and then dried with calcium hydride. The final product was 22.5 g with a yield of 61.7%. The following were obtained: ^{1}H NMR (500 MHz, $CDCl_3$): δ ppm, 0.8 (d, 6H), 2.17 (m, 1H), 3.04 (t, 2H), and 3.83 (t, 2H).

2.4 Synthesis of compound POx

The synthetic procedure was followed as reported previously in the literature.[S6] Under the protection of nitrogen, methyl p-toluenesulfonate (0.21 mL, 1.37 mmol) and Ox (10 mL, 84 mmol) were mixed and dissolved in 15 mL anhydrous acetonitrile, and finally reacted at 40 ^{0}C for 3 days until the reaction solution turned slightly yellow. After cooling to room temperature and adding 200 mg sodium azide for 2 days, the reaction solution was dialyzed for 3 days. The target product **POx** was obtained by freeze-drying. The yield was 46.8%. The following were obtained: ^{1}H-NMR (400 MHz, $CDCl_3$) δ (ppm): 3.44 (broad s; -CH_2 on the polymer backbone), 3.06 (s; terminal -CH_3), 2.94 - 2.64 (two broad s; -CH on the polymer side chain), and 1.10 (broad s; -CH_3 on the polymer side chain).

2.5 Synthesis of F1-POx

The synthetic procedure was appropriately modified according to data previously reported in the literature.[S7] **F1** (20 mg), **POx** (600 mg) and cupric sulfate pentahydrate (56 mg) were mixed and dissolved in 5 ml THF. Sodium ascorbate (45 mg) was dissolved in 1 mL of water and poured into the above mixed solution. The reaction solution was stirred and refluxed for 2 days. After the reaction completed, the filtrate was filtered and lyophilized to obtain the brown solid product with a yield of 78%. The following were obtained: ^{1}H-NMR (500 MHz, $CDCl_3$) δ (ppm): 7.15-6.90 (m; -CH- in phenyl ring of TPE unit), 5.42-5.37 (m, -CH_2- near phenyl ring of TPE unit), 4.65-4.52 (m, -CH_2- in triazole ring and -CH_2- in polymer backbone near triazole ring), 3.61 (broad s, -CH_2- of the polymer backbone), 2.94-2.64 (br, -CH- of the polymer side chain), and 1.33 (strong br, -CH_3 on the polymer side chain).

2.6 Synthesis of 2-(2-hydroxy-3-methoxyphenyl) benzoxazole (2)

The compound was synthesized according to the appropriate modification of our

work reported previously.[S8] *o*-Vanillin (1.0 g, 6.6 mmol) and *o*-aminophenol (0.72 g, 6.6 mmol) were dissolved in methanol (35 mL) and heated to 45 ^{0}C for 12 h under the protection of nitrogen gas. The residue was dissolved in 10 mL dichloromethane, and into which 1.49 g dichlorobenzoquinone (DDQ) dissolved in 200 mL dichloromethane solution was added. The mixture was stirred at room temperature for 3 h. The reaction mixture was washed with 100 mL saturated sodium carbonate solution, water, saturated NaCl aqueous solution in turn and dried with anhydrous sodium sulfate. The crude product was purified by rapid column chromatography (eluent: petroleum ether/dichloromethane = 2:1) and then crystallized with absolute ethanol to yield 0.7 g white crystal with a yield of 41.0%. The following were obtained: ^{1}H NMR (500 MHz, $CDCl_3$) δ (ppm), 7.79 (dt, J = 5.4, 3.3 Hz, 1H), 7.73-7.61 (m, 2H), 7.47-7.37 (m, 2H), 7.08 (dd, J = 8.0, 1.3 Hz, 1H), 7.00 (t, J = 8.0 Hz, 1H), and 4.01 (s, 3H).

2.7 Synthesis of 2-(2,3-bihydroxyphenyl)benzoxazole (3)

Compound **2** (0.5 g, 2.1 mmol) and 30 mL of dry dichloromethane were added to a 100 mL round-bottom flask cooled in an ice-salt bath. A total volume of 20 mL dry dichloromethane was added, and then 0.8 mL (8.3 mmol) of boron tribromide was added dropwise under nitrogen protection. The resulting solution was further stirred at room temperature for 24 h. After reaction, 40 mL ice water was added to quench the reaction solution. The precipitated solid was collected through filtration. The liquid phase was extracted with dichloromethane three times. The organic phase was washed with saturated NaCl aqueous solution and distilled water. Finally, the product was dried by anhydrous sodium sulfate and the solvent was removed by rotary evaporator under reduced pressure. The solid product (0.35 g) was obtained by crystallizing with ethanol as a white solid with a yield of 70%; ^{1}H NMR (500 MHz, $CDCl_3$) δ ppm, 7.81-7.76 (m, 1H), 7.69-7.64 (m, 1H), 7.61 (dd, J = 8.0, 1.4 Hz, 1H), 7.47-7.41 (m, 2H), 7.15 (dd, J = 7.9, 1.4 Hz, 1H), 6.98 (t, J = 8.0 Hz, 1H), and 5.84 (s, 1H).

2.8 Synthesis of 2-(2-hydroxy-3-propargyl phenyl) benzoxazole (F2)

Compound **3** (0.12 g) was dissolved in 20 mL anhydrous acetone. Potassium carbonate (0.12 g) was added and stirred for 1 h at room temperature. 3-bromopropyne (37 mL) was added to the reaction solution. The mixture was stirred and refluxed for 24 h. After reaction, the solvent was removed and the residue was purified by silica gel column chromatography to afford **F2** as a yellow-white powder (eluent: petroleum ether/dichloromethane = 2:1); m.p. 195.5 °C. The following were obtained: ^{1}H NMR (500 MHz, $CDCl_3$) δ (ppm), 11.73 (s, 1H), 7.77 – 7.70 (m, 2H), 7.64 – 7.58 (m, 1H), 7.41 – 7.36 (m, 2H), 7.23 (dd, J = 8.0, 1.4 Hz, 1H), 6.99 – 6.90 (m, 1H), 4.87 (d, J = 2.4 Hz, 2H), 2.54 (t, J = 2.4 Hz, 1H). ^{13}C NMR (126 MHz, DMSO-d_6) δ (ppm): 182.53, 176.15, 170.14, 160.15, 140.13, 135.52, 131.28, 129.64, 129.51, 127.02, 123.81,

122.65, 53.84, 38.01, 37.36. HRMS(ESI) m/z: calcd. for $C_{16}H_{12}NO_3$, 266.0817 $[M+H]^+$, and found 266.2692.

2.9 Synthesis of sensor F2-POx

F2 (20 mg), **POx** (800 mg) and cupric sulfate pentahydrate (56 mg) were mixed and dissolved in 5 mL THF. Sodium ascorbate (45 mg) was dissolved in 1 mL water and poured into above mixture. The reaction solution was stirred and refluxed for 2 days. After filtration, the solid product as coffee liquid was obtained after dialysis. Yield: 65%. ^{1}H-NMR (500 MHz, $CDCl_3$) (ppm): 10.44 (s; -OH of Benzoxazole), 7.66-6.76 (m, Ar-H of Benzoxazole), 4.77-4.89 (m, $-CH_2-$ in triazole ring and $-CH_2-$ in polymer backbone near triazole ring), 3.44 (br, $-CH_2-$ of the polymer backbone), 2.90-2.64 (br, -CH of the polymer side chain), and 1.41 (strong br, $-CH_3$ of the polymer side chain).

3. Supporting Figures

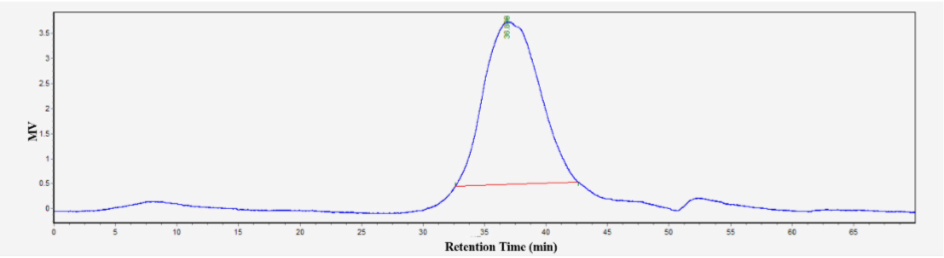

Fig. S4. GPC chromatogram of **F1-POx**.

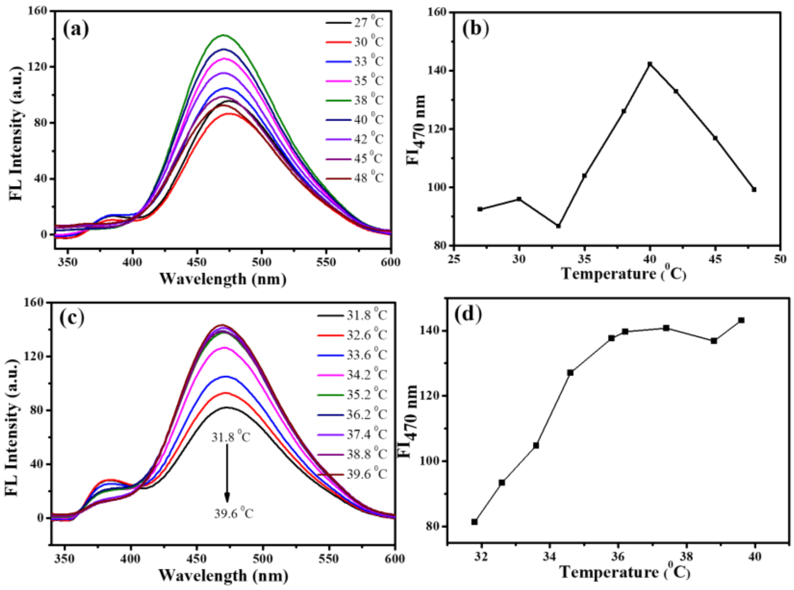

Fig. S5. (a, c) Fluorescence spectra of **F1-POx** in PBS buffer (10 mM, pH 7.4) at various temperatures, λ_{ex}=305 nm, the temperature increases at 2-3 ^{0}C (a) and 0.6-1.4 ^{0}C (b) intervals. (b, d) Fluorescence intensity of **F1-POx** at 470 nm vs. temperature in PBS buffer (10 mM, pH 7.4); here, (b) and (d) correspond to (a) and (c), respectively.

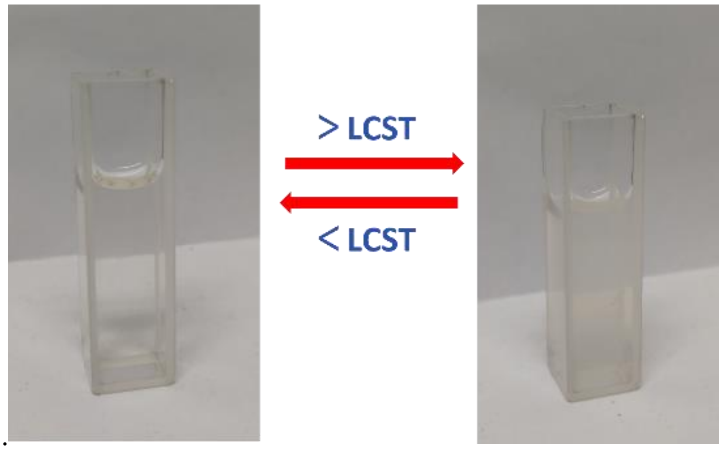

Fig. S6. Photos of **F1-POx** in aqueous solution in the presence of cyclodextrin at 25°C (left) and 50°C (right).

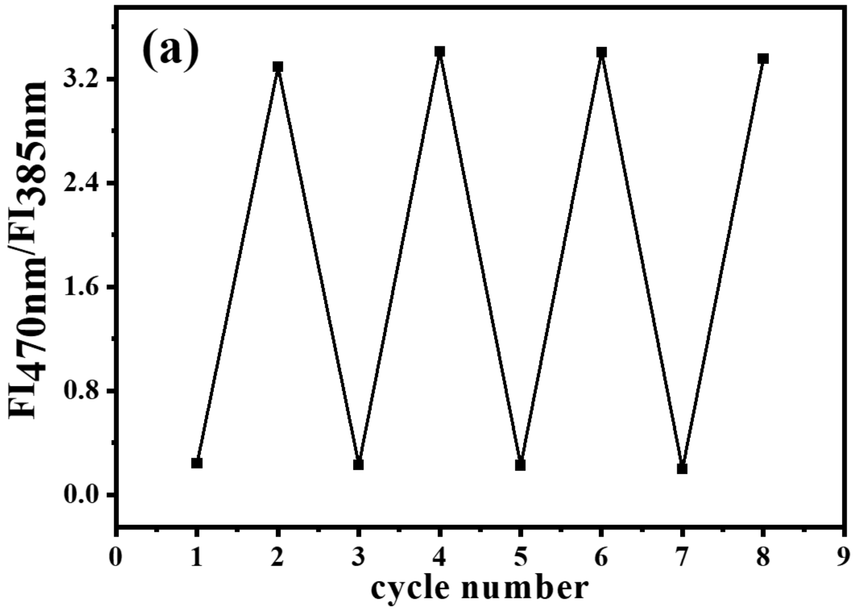

Fig. S7. Fluorescence intensity ratio changes of **F1-POx** and excess γ-CD upon repeated heating and cooling cycles at 36 ^{0}C and 41 ^{0}C.

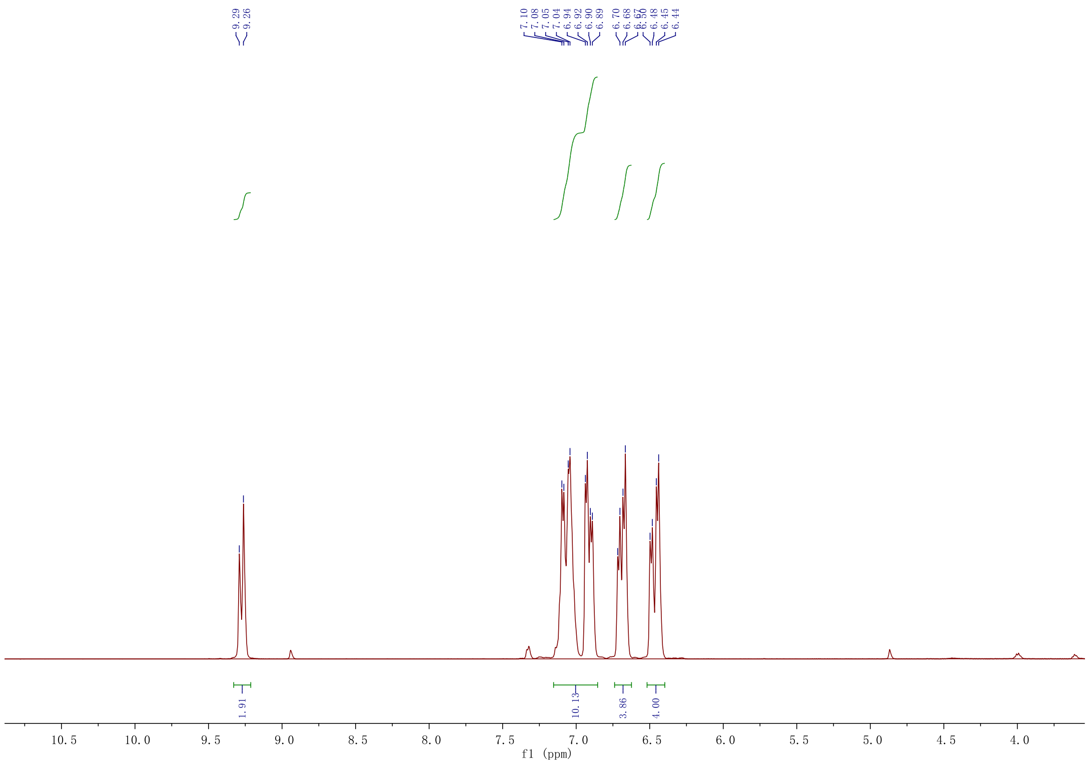

Fig. S8. ^{1}H NMR spectrum of **1** in DMSO-*d*6.

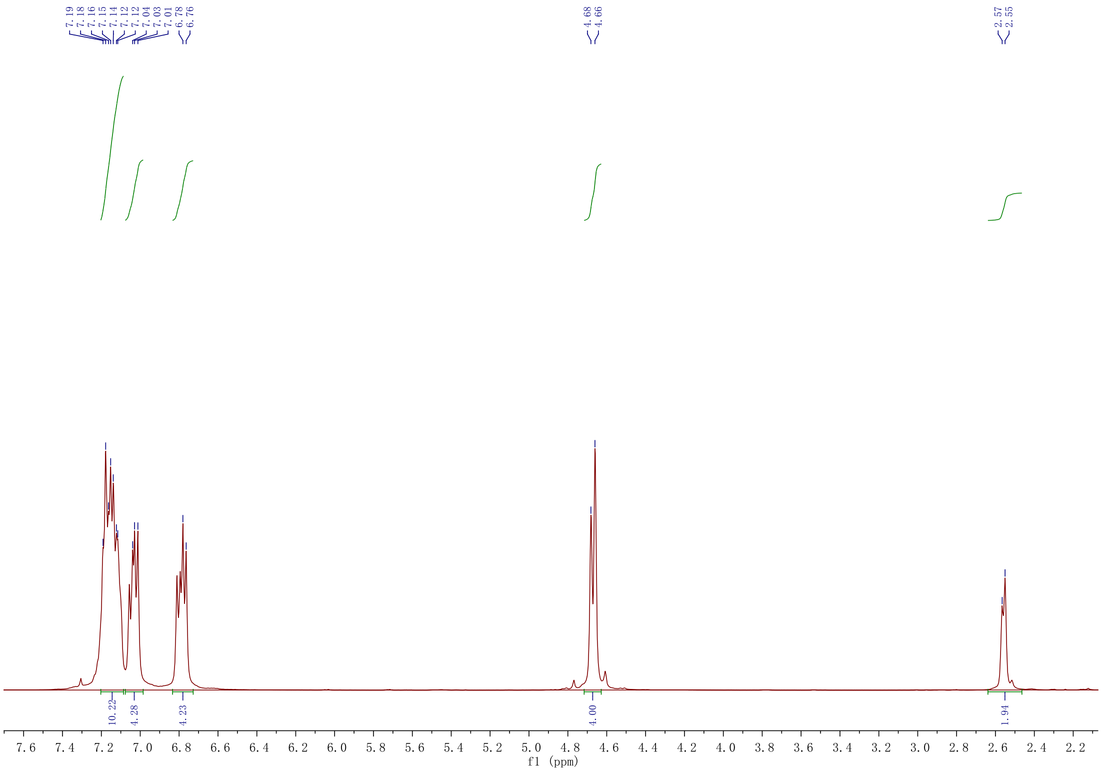

Fig. S9. ^{1}H NMR spectrum of **F1** in DMSO-*d*6.

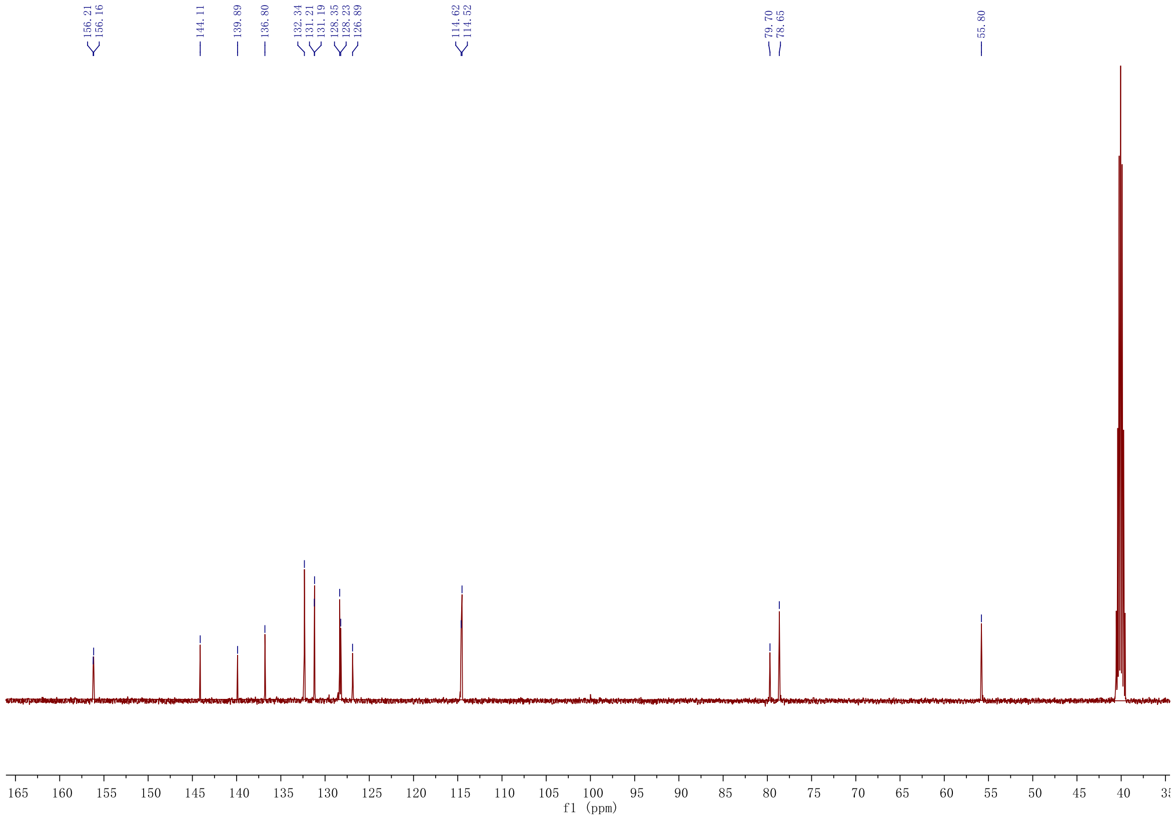

Fig. S10. ^{13}C NMR spectrum of **F1** in DMSO-d_6.

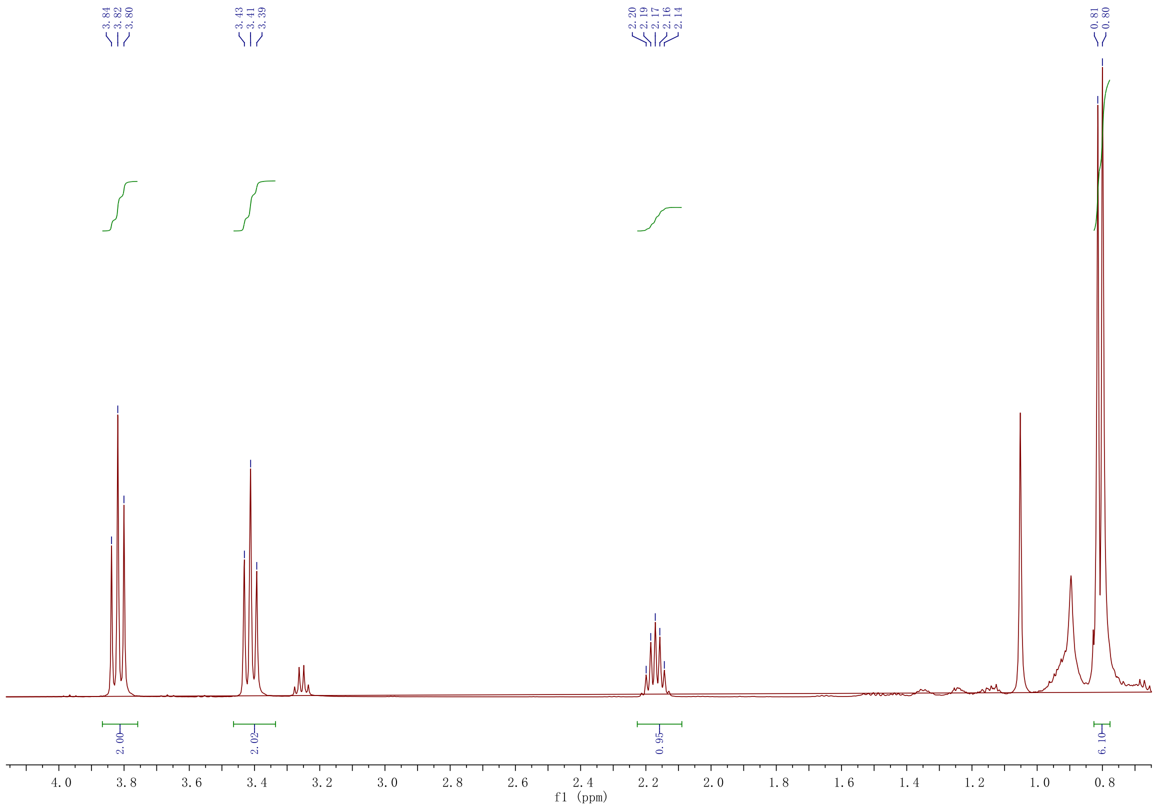

Fig. S11. ^{1}H NMR spectrum of **Ox** in $CDCl_3$

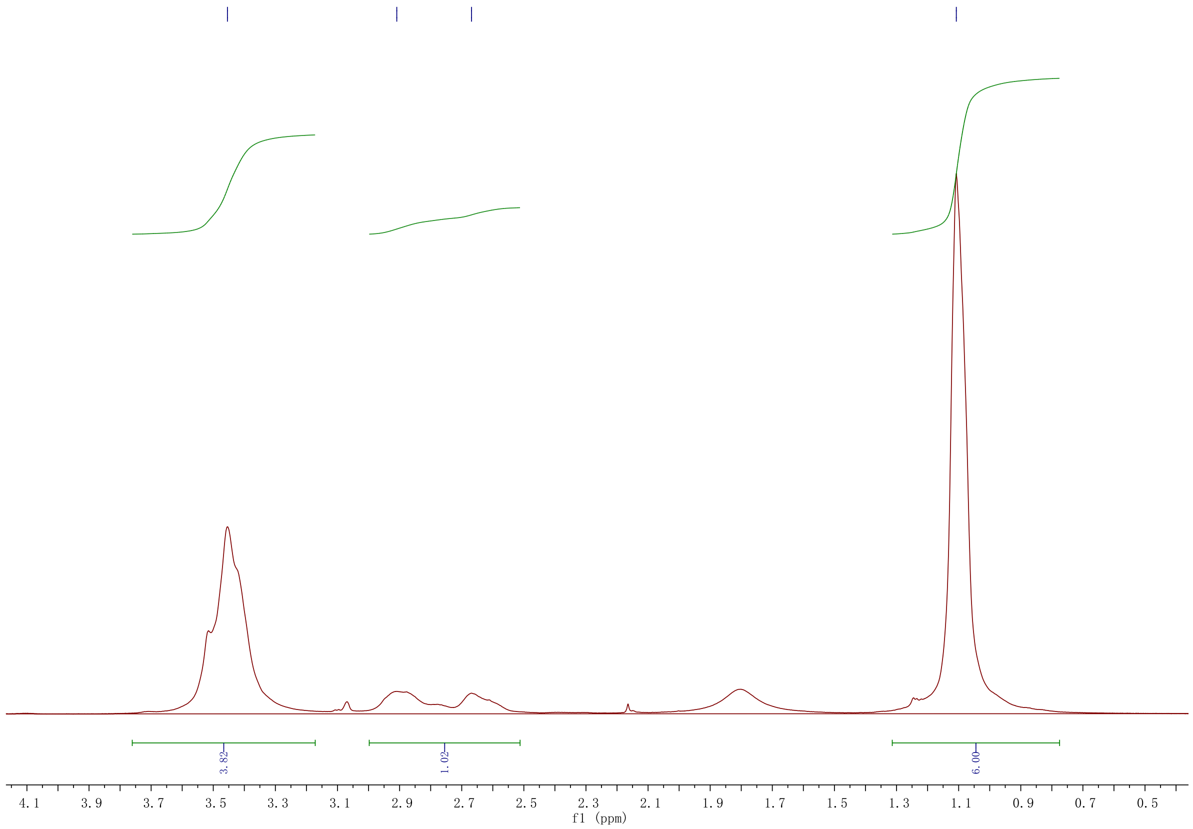

Fig. S12. ^{1}H NMR spectrum of **POx** in $CDCl_3$.

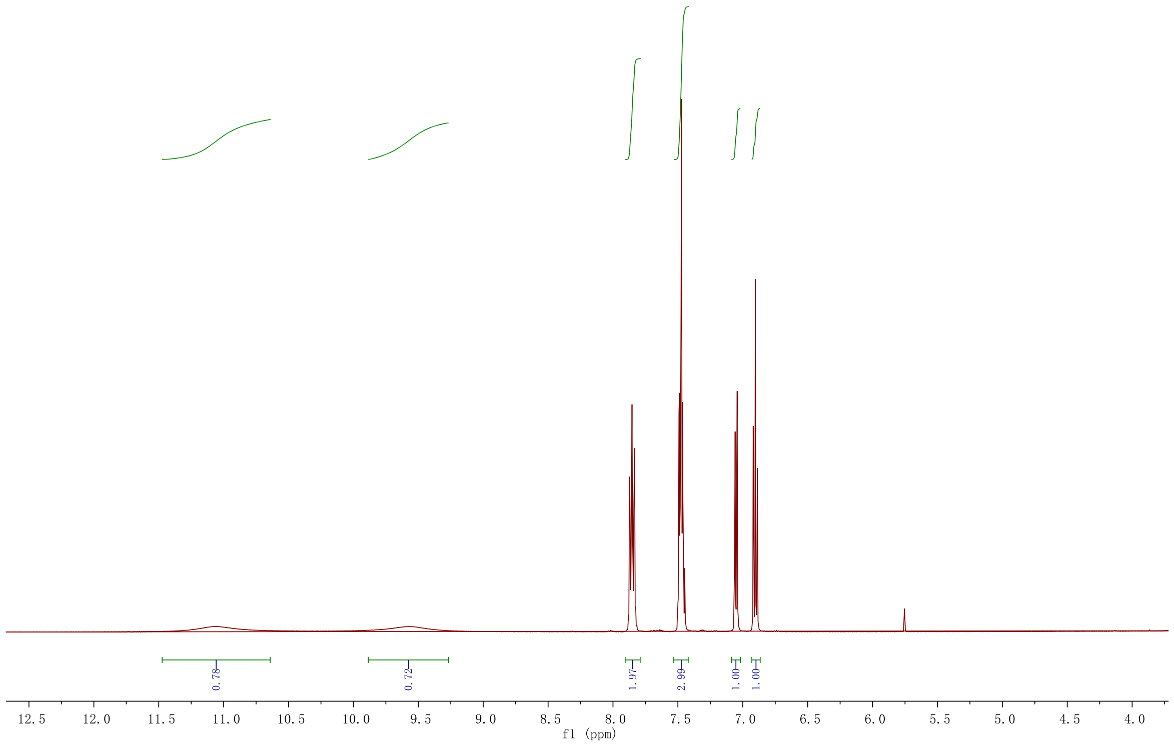

Fig. S13. ^{1}H NMR spectrum of **3** in DMSO-d_6.

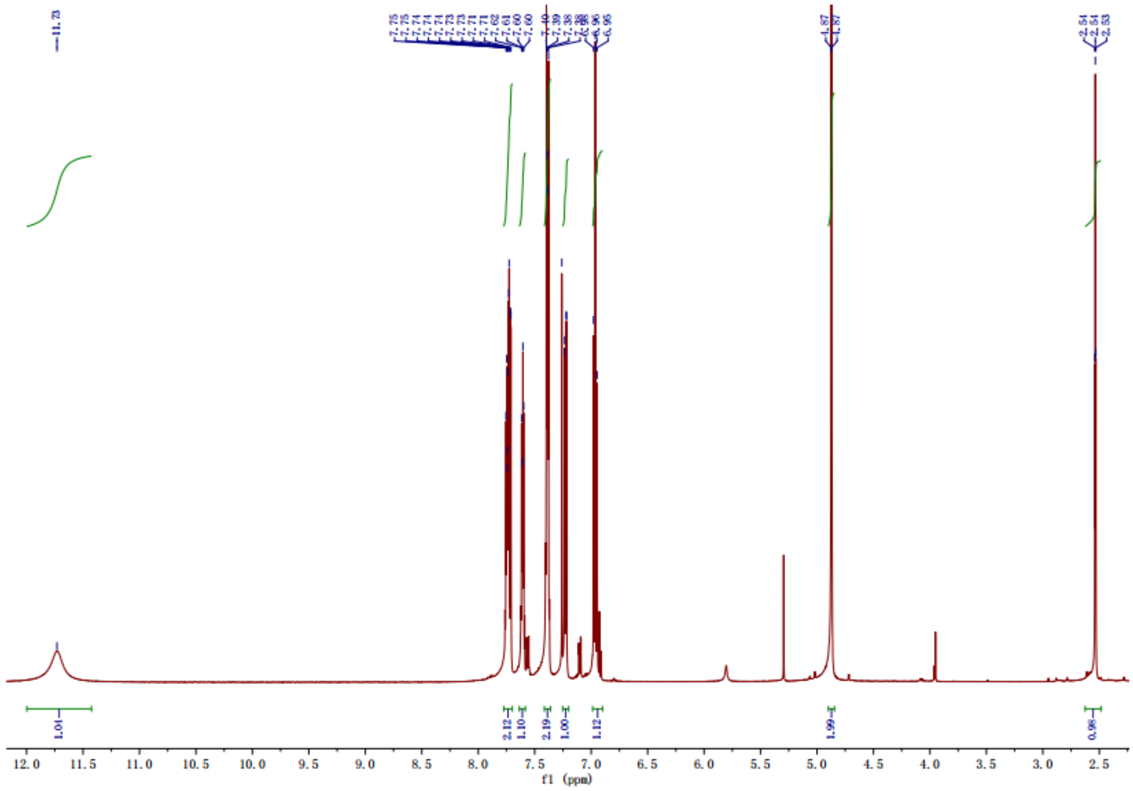

Fig. S14. ^{1}H NMR spectrum of **F2** in $CDCl_3$.

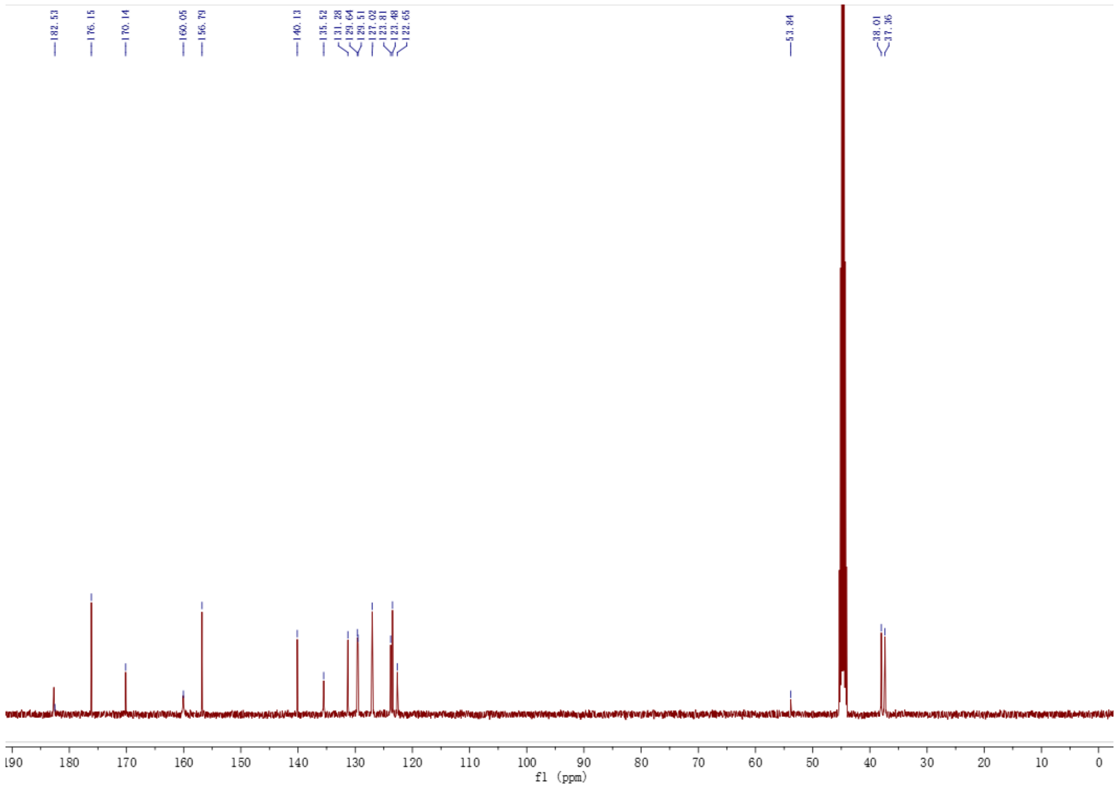

Fig. S15. ^{13}C NMR spectrum of **F2** in DMSO-d_6.

References:

S1. S. Uchiyama, C. Gota, T. Tsuji and N. Inad, *Chem. Commun.*, **2017**, 53, 10976-10992.

S2. A. Halperin, M. Kröger and F. M. Winnik, *ACS Appl. Polym. Mater.,* **2024**, 21, 13202–13209.

S3. W. Guan, W. Zhou, C. Lu and B. Z. Tang, *Angew. Chem. Int. Ed.,* **2015**, 54, 15160-15164.

S4. Q. Chen, N. Bian, C. Cao, X.-L Qiu, A.-D. Qi and B.-H. Han, *Org. Biomol. Chem.,* **2019**, 17, 9251-9256.

S5. M. Meyer and H. Schlaad, *Macromolecules*, **2020**, 53, 5056–5067.

S6. J.-H. Kim, Y. Jung, D. Lee and W.-D. Jang, *Adv. Mater.*, **2016**, 28, 3499-3503.

S7. J.-H. Kim, D. Yim and W.-D. Jang, *Chem. Commun.*, **2016**, 52, 4152-4155.

S8. Y. Xu, L. Xiao, S. Sun, Z. Pei Y. Pei and Y. Pang, *Chem. Commun.*, **2014**, 50, 7514-7514.
